# Anorectal mucosal melanoma

**DOI:** 10.18632/oncotarget.23835

**Published:** 2018-01-02

**Authors:** Giulia Malaguarnera, Roberto Madeddu, Vito Emanuele Catania, Gaetano Bertino, Luca Morelli, Rosario Emanuele Perrotta, Filippo Drago, Michele Malaguarnera, Saverio Latteri

**Affiliations:** ^1^ Research Center "The Great Senescence", University of Catania, Catania, Italy; ^2^ Department of Biomedical Sciences, University of Sassari, Sassari, Italy; ^3^ Department of Medical, Surgical Sciences and Advanced Technologies “G.F. Ingrassia”, University of Catania, Catania, Italy; ^4^ Hepatology Unit, Department of Clinical and Experimental Medicine, University of Catania, Policlinico "G. Rodolico", Catania, Italy; ^5^ Department of Surgery, University of Pisa, Pisa, Italy; ^6^ Department of General Surgery and Medical-Surgery Specialties, University of Catania, Catania, Italy; ^7^ Department of Biomedical and Biotechnological Science, University of Catania, Catania, Italy

**Keywords:** malignant melanoma, mucosal melanoma, anorectal tumours, anorectal melanoma, treatment

## Abstract

Anorectal melanoma is an uncommon and aggressive mucosal melanocytic malignancy. Due to its rarity, the pre-operative diagnosis remains difficult. The first symptoms are non-specific such as anal bleeding, anal mass or pain. Although anorectal melanoma carries a poor prognosis; optimal therapeutics strategies are unclear.

Surgical resection remains the mainstay of treatment. The optimal surgical procedure for primary tumours is controversial and can vary from wide local excision or endoscopic mucosal resection (EMR) to an abdomino-perineal resection.

A high degree of uncertainly exists regarding the benefit of radiation therapy or chemotherapy. The treatment of advanced melanoma is evolving rapidly with better understanding of the disease biology and immunology. Considerable effort has been devoted to the identification of molecular determinants of response to target therapies and immunotherapy.

## INTRODUCTION

Anorectal malignant melanoma (ARMM) is a rare and highly malignant tumor with less than 5 year survival of 10% patients [1, 2].

ARMM is often misdiagnosed in about two thirds of patients and most often as haemorrhoids, adenocarcinoma polyps and rectal cancer [3]. The typical symptoms such as anal pruritus or rectal pain can mimic haemorrhoids or rectal polyps [4]. ARMM can be confused with lymphoma, anorectal carcinomas or sarcoma because of its rarity and histological variability [5].

Comparing ARMM with other anorectal carcinomas, tumour cells can exhibit multiple cell types characteristics [6, 7].

The poor knowledge about aetiology, pathogenesis and genetics of ARMM makes difficult proper diagnosis, treatment and prognosis.

### Epidemiology

ARMM accounts for only 1.3% of all melanomas and 16.5% of mucosal melanomas [2]. The incidence of ARMM increases with age and the prevalence is 1.6 to 2.3 times higher in women than in men. Moreover, it has been reported that ARMM is twice as prevalent in Caucasian as in African Americans [8–10].

Lesions can affect anal canal, rectum or both, but the great majority of tumours are located with in 6 cm of the anal rim [11].

Approximately 20–30% of ARMMs are amelanotic, can endoscopically resemble benign polypoid lesions, and unspecific symptoms can contribute to misdiagnosis [12, 13].

Amelanotic lesions are frequent in mucosal melanoma [14]. The incidence is difficult to calculate because several authors indicate amelanotic melanomas those only partially devoid pigment at visual inspection [15–17].

Amelanotic ARMMs can present some pigmentation at the periphery of the lesion and it is characterised by a worse prognosis because of the difficulty of diagnosis and the invasive nature [18, 19].

### Embriology of anorectal melanomas

The anal canal is derived from the hindgut proximally by the upper limit of the anal transition zone (ATZ) and the ectodermal proctodeum distally with the dentate line at the fusion point [7, 20].

Melanocytes arise from neuro-ectodermal multipotent neural crest cells [21].

Melanocytes can be found in the anal squamous zone, sometimes in the anal transitional zone and never in the colorectal zone. These potential cells migrate via the umbilical-mesenteric canal and later differentiate into specialized cells, which undergo neoplastic transformation. According to this theory, ileum that represents the distal end of the umbilical mesenteric canal should be the commonest of primary malignant melanoma of the small intestine [22]. However, benign melanocytes had been found not only in the epithelium area around resected primary anal melanomas, but also in the squamous, in transitional zone, and in the colorectal zone.

### Physiopathology of melanocyte

Melanocytes are melanin-producing somatic cells, which provide physiological functions [23].

Melanin plays a key role in protection from stressors such as UV radiation, acting as sunscreen, or as metal ion and radical scavenger, energy transducer and also it can bind organic molecules and some drugs. Melanocytes in mucosal membranes are involved not only in antimicrobial defence, but also in the protective role of immune response and probiotics that may interact with the host [24, 25]. Immune modulation is due to the release of IgA, cytokines and both Natural Killers and Dendridic cells activity [26]. Beyond modulation of immune response, other mechanisms include the increase in barrier function, antagonism of pathogens, and production of substances.

In gastrointestinal barrier, melanocytes are important for structural changes in the epithelium, promoting the formation and the redistribution of some protein, in order to support the physical barrier; whereas in the mucous barrier, melanocytes induce mucin production in the Globet cells and up-regulates defensin production in the Paneth cells [27, 29].

There are evidences supporting other non pigment-related functions of melanocytes such as antimicrobial and immunologicalmetabolic, endocrine, chemical and physical functions [28].

Melanin is produced within membrane-bound organelles (melanosomes), which are transferred to the dendrites and eventually phagocytized by the keratinocytes. Melanoblasts, precursor cells to melanocytes, migrate during foetal development, throughout the dermis to the skin, the eye, the inner ear and the leptomeninges [29]. The pigmentation degree of melanin varies between people of different racial and ethnic groups and also between same populations [30, 31].

Melanocytes in the ano-genital give rise to melanomas that have a low mutation burden but highly rearranged genomes with numerous copy number changes, including multiple focused amplifications.

Melanosomes are organelles of melanocyte important not only for melanin biosynthesis, but also because they contain proteins and enzymes useful to maintain melanosome structure and to the maturation of the immature pre-melanosome [32].

### Pathogenesis

The pathogenesis of anorectal melanomas is limited and obscure.

There are several theories for the origin of malignant melanoma in the gastro-intestinal tract. Although anorectal contains no melanocytes, these cells have been occasionally found in the alimentary tract. Some authors suggest that malignant melanoma develops from intestinal Schwann cells. Other authors proposed that malignant melanomas originate from neural crest and during embryogenesis migrated to the basal layer of the epidermis and the hair follicles [19, 20, 33].

### Diagnosis

The diagnosis of ARMM remains difficult for the non specificity of the symptoms and the diagnosis is frequently made after treatment for presumed benign disease, such as hemorroidectomy.

The evaluation of the primitivity include presence of single lesion, absence of other primary site melanoma, no history of removal of melanoma or atypical melanocytic lesions, the presence of lymphocytes infiltration surrounding tumour mass [34] and absence of enlargement of draining lymph nodes, and survival time over on year after the diagnosis [35].

Blecker *et al.* [36] added in the diagnosis criteria the presence of intra mucosal melanocytic lesions in the overlying or adjacent intestinal epithelium.

### Symptoms

Symptoms prior to diagnosis are similar to those caused by haemorroids, adenocarcinama polyps, rectal cancer, such as:

1. elimination of mucus and blood through the anal canal
2. anal pain or discomfort, tenesmus
3. feeling of rectal fullness or incomplete evacuation
4. externalization of tumour and changes in bowel habits
5. pruritus
6. changes in bowel movements
7. inguinal masses

The diagnosis of an ARMM is made by biopsy and immunohistochemical staining. Lesions detected at colonscopy were characterized for morphology, appearances of lesions (including margins, colorations, origins, surface and invasion of dentate line characteristics) and presence of superficial melanin pigmentation. The use of colonscopy and endoscopic ultrasound (EUS) is helpful for diagnosis and staging of ARMM. Colonscopy combined with biopsy and subsequent pathological examination allows the accuracy in the ARMM diagnosis [37, 38].

Histological and immunochemistry are the gold-standard diagnostic method. Histological examination characterises the lesions: cell type, degree of melanin pigmentation, mitotic index.

Mucosal melanomas shows high pleomorphism in the nucleus, epithelioid spindle-shaped and often they present melanin granules [21].

In other cases, positivity for carcinoembryonic antigen, CD30 and CD68 can also be found as well as negativity to AE1/AE3, CD 17 and desmin [39].

Other histological criteria are the proliferation of atypical junctional melanocytes and atypical melanocytic cells in the basal layer in the superficial epithelium.

### Immunochemical and molecular profile of anorectal melanoma

In ARMM melanocytes can appear in several forms (pleomorphic, epithelioid, spindle cells, etc), creating complication in the differential diagnosis with other tumors such as sarcomas, gastrointestinal tumour (GIST) and undifferentiated carcinomas.

Immunohistochemical diagnosis is possible thanks to protein S-100, HMB-45, Melanin A and Mart-1 antibodies.

Identification of multiple oncogenes and development of small molecular target therapies against melanomas had been possible thanks to the recent discoveries about this desease [40].

The immunohistochemical staining for cKIT (CD117) protein, a type III transmembrane tyrosine receptor, has become essential for diagnosis of Gastrointestinal stromal tumours (GISTs) and also for the differential diagnosis of mesenchymal tumours of gastrointestinal tract [41, 42].

cKit includes five distinct domains: 1) a glycosilated extracellular ligand binding domain, 2) a hydrophobic transmembrane domain, 3) an intracellular juxtamembrane domain and 4) two tyrosine kinase domains [43]. Kit is a proto-oncogene that encodes c-kit protein (CD117) which is a transmembranous receptor kinase target of imatinib. Mucosal melanomas are likely to harbour higher rates of kit mutations when compared with other melanoma subtypes [44]. Kit Mutation was reported in 35.5% of ano-rectal melanomas [45] Not all tumors with KIT gene alterations are immunopositive for CD117 and its mutation frequency is high in ARMM [46].

KIT activation triggers a variety of downstream pathways, including MAPK/MEK and PBK/AKT pathways, which may play an important role in the development of melanoma. In samples of anorectal melanoma patients Hintzsche identified in 4/5 KIT and NF1 mutation in 3/5 SF3B1 R625H/S/C and NRAS mutation and in 2/5 BRAF mutations [47]. However, considering the rarity in observing the c-Kit gene mutations in the European population, is given a preliminary assessment of the status of the BRAF gene mutation and NRAS, prior to the determination of mutations in c-Kit. Melanomas with aberrations of the KIT genes might represent another subgroup, which benefit from a therapy targeting the gene product c-Kit.

BRAF, NRAS and Kit mutations stand out in pathogenesis and targeted therapy of melanoma. BRAF and NRAS both take part in the mitogen-activate protein kinase (MAPK) pathway which significantly contributes towards melanoma development [48, 49]. The frequency of mutations of the BRAF gene in anorectal melanoma is lower than that of cutaneous melanomas, while the c-Kit gene would prevail (39%). Maldonado JL found BRAF mutations in 2/21 mucosal melanomas [50], Cohen Y in 1/25, whereas Edwards RH found none BRAF mutation in 13 mucosal melanomas [51, 52].

### Serum markers in melanoma

No findings are present in literature regarding molecular markers that enable melanocytes to anorectal melanoma, but several serum tumour markers may be investigated.

LDH is a standardized tumour marker, characterized by poor specificity and it is very useful for detecting distant metastases [53]. LDH in the serum of patients have been found in advances melanoma when part of tumour outgrows its blood supply. Since is not a secreted enzyme, the spillage of LDH probably occurs during apo-necrosis or melanoma-cells necrosis.

Other serum tumour markers are S-100B, melanoma inhibitor activity protein (MIA) Enolase, tumour associated antigen 90 immune complex and more recently YKL-40) [54–58].

S-100B is a 21 Kda protein that was first isolated by Moore from the central nervous system of vertebrates [59], chiefly found in glial and Schwann cells and in particular in cell cytoplasm and membrane. The name is derived from the protein solubility characteristic: 100% in saturated ammonium sulphate at neutral pH. S-100B is a dimer, consists of two isomers a and b; all possible combination can occur (S-1001a, S-100 ab, and S-100bb) [60, 61].

S-100B has calcium-binding properties and it inhibits protein phosphorylation and cytoskeleton formation, which may favour tumour progression.

The S-100B protein originated from neuroectoderma and mesoderma is expressed in various part of the body [62] and is involved in cytoskeletal regulation, playing a possible role in cell cycle progression.

MIA is an 11-K Da soluble protein strongly expressed by malignant melanocytes. MIA, as an autocrine tumour cell growth inhibitor, decreased cell attachment tumour metastases. In normal skin MIA shows low or no expression, while naevi show only moderate, low or no expression [63], MIA gene locus has been mapped to chromosome 19q13.32 in humans and its DNA sequence has been fully described [64]. Serum levels of MIA seem to reflect tumor burden in some patients with metastases. Moreover, in regional lymph node metastases or distant metastases MIA and S-100B had been found present and their elevations have been associated with a poor prognosis.

Enolase is a dimeric enzyme of the glycolytic pathway that consists of three possible subunits: alfa, beta and gamma [65]. The alfa-gamma and gamma-gamma dimers are found in neurones and neuroendocrine cells and then they are called neuroenolase (NSE), which levels have a higher predictive value in melanoma. Positive serum NSE levels indicated progression of disease [66].

MoAb-079 and NSE may be less sensitive markers than P-S100 and HMB-45 for routinely processed mucosal melanomas as compared with metastatic tumours.

YKL-40 is a 40 kDa heparin and chitin-binding lectin, member of the chitinase-like protein family. YKL-40 is a glycoprotein involved in neo-angiogenesis, inflammation and reconstruction of extracellular matrix, secreted by different cells such as macrophages, chondrocytes or vascular smooth cells [67, 68]. In advanced metastatic melanoma, YKL-40 has been correlated with the site of metastases and poor performance status as well as with overall survival [69].

YKL-40 play an important role in neo-angiogenesis acting as a potent angiogenic factor: it stimulates tumour vascularisation and induces FAK-MAPK via up-regulation of VEGF receptor 2 in endothelial cells. YKL-40 stimulates also endothelial cells to migration showing similar activity as VEGF [70].

Recent studies are investigating the YKL-40 mechanisms of action, as well as its utility in anti- YKL-40 therapies [71] such us Interleukin-2 and interferon α [72, 73].

### Imaging findings

Multiple imaging diagnostic are used in ARMM to evaluate primary cancer, metastasis and treatment responses [22]. Ultrasonography (US), Endoscopic Ultrasound (EUS), Computed Tomography (CT) Magnetic Resonance (MRI), Positron emission tomography (PET) contribute to the information for diagnosis and management. US is helpful to differentiate between solid and cystic lesions in the liver. EUS examination is useful to evaluate lesion size, invasion level and depth within and beyond the bowel wall. EUS showed lesions as masses originating in the mucosa with inhomogenous or low-level internal echoes. Lesions exhibited irregular margins and degrees of submucosal infiltration [38]. CT can exhibit hepatic and pulomonary metastasis and shows enhancement during the late arterial phase and hypoattenuation of liver parenchyma in the portal venous phase [74, 75].

PET/CT is recommended in staging and response assessment of metastatic melanoma. Malignat cells have greater FDG avidity than adjacent normal tissues because of their higher metabolic rate. Therefore PET/CT allow evaluation of site of metastasis and may help in staging disease and the therapy. MRI is advantageous for preoperative staging in anorectal melanoma patients and for detection of metastatic lesions, in particular in those patients who have hepatic metastases bowel wall invasion. Similar to other sites anorectal melanomas are hyperintense on T-1 weighted images; however, amelanocitic melanomas, which comprise approximately 10 to 30% of anorectal melanomas, are hypointense on T1 and hyperintense on T2 weighted sequences [76].

### Staging

Anal melanoma is staged in: stage I (local disease), stage II (local disease with regional lymph nodes), and stage III (with distant metastasis) [77, 78]. Prasad ML [79] *et al.* classified mucosal melanomas in Level 1 (local or *in situ* tumor); level 2 (regional with invasion of lamina propria), level 3 (disseminated) [21]. In stage 1 tumour growth is limited to the bowel wall or anal skin; in stage 2 disease involves regional lymph nodes metastases; in stage 3 the tumour extend beyond the surgical resection margin. Most patients with distant metastases have hepatic metastases, followed by pulmonary and bone metastases.

There are differences concerning survival for the three groups. The median survival was 138 months for level 1,9 months for level 2 and 17 months for level 3. The disease is often first diagnosed at its advanced stages, and for this reason probably the prognosis is poor.

### Treatment

**Figure 1:**
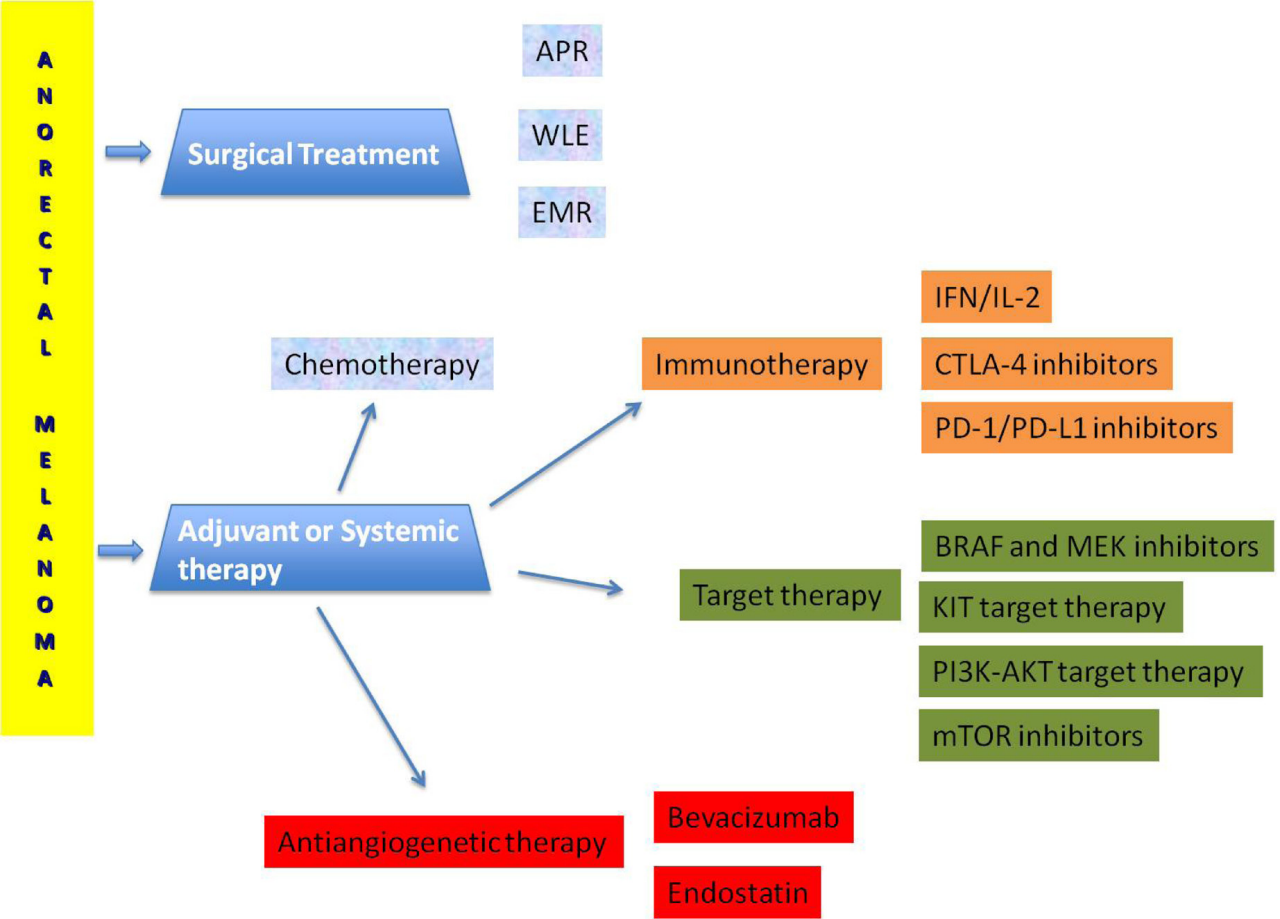
Terapeutic management of anorectal melanoma

The management strategies on melanoma control and the consequent survival is difficult because of the absence of randomised trials. Surgery, radiotherapy, chemo-immunotherapy and targeted therapy provide uncertain results (Figure 1).

### Surgery treatment

The typical treatment is surgical resection; however, standard operative procedures related to the area of resection and lymph dissection have yet to be established. The presence or absence of lymphatic metastasis has not demonstrated any significant difference in prognosis.

The conditions for ensured long-term survival are:

- Maximum tumour diameter <5 cm
- Wall invasion depth within the muscularis propria
- Extensive lymph node dissection regardless of the presence or absence of lymphatic metastases [80].

Surgery was defined as R0 when the tumor margins were microscopically and macroscopically negative, R1 when the margins were positive on microscopy, and R2 when resection were macroscopically incomplete. Surgical treatments include abdominoperineal resection (APR), wide local excision (WLE) and endoscopic mucosal resection (EMR). EMR in some cases remove melanoma with long-term survival [81]. WLE has minimal morbidity and no compromise local function, preserve the anal sphincter. APR is often associated with high morbidity rate and functional compromise [82–84]. The small number of studies shows no significant differences in survival between patients treated with APR and WLE [85]. WLE was recommended whenever possible as initial and limited treatment, whereas patients undergoing to APR were less likely to develop local recurrences.

Many surgeons perform WLE followed by adjuvant radiotherapy to the pelvis and inguinal lymph nodes in most patients due to the similar local control rate as APR, which is preferred in the cases with local extensive disease not amenable to a local excision.

**Figure 2:**
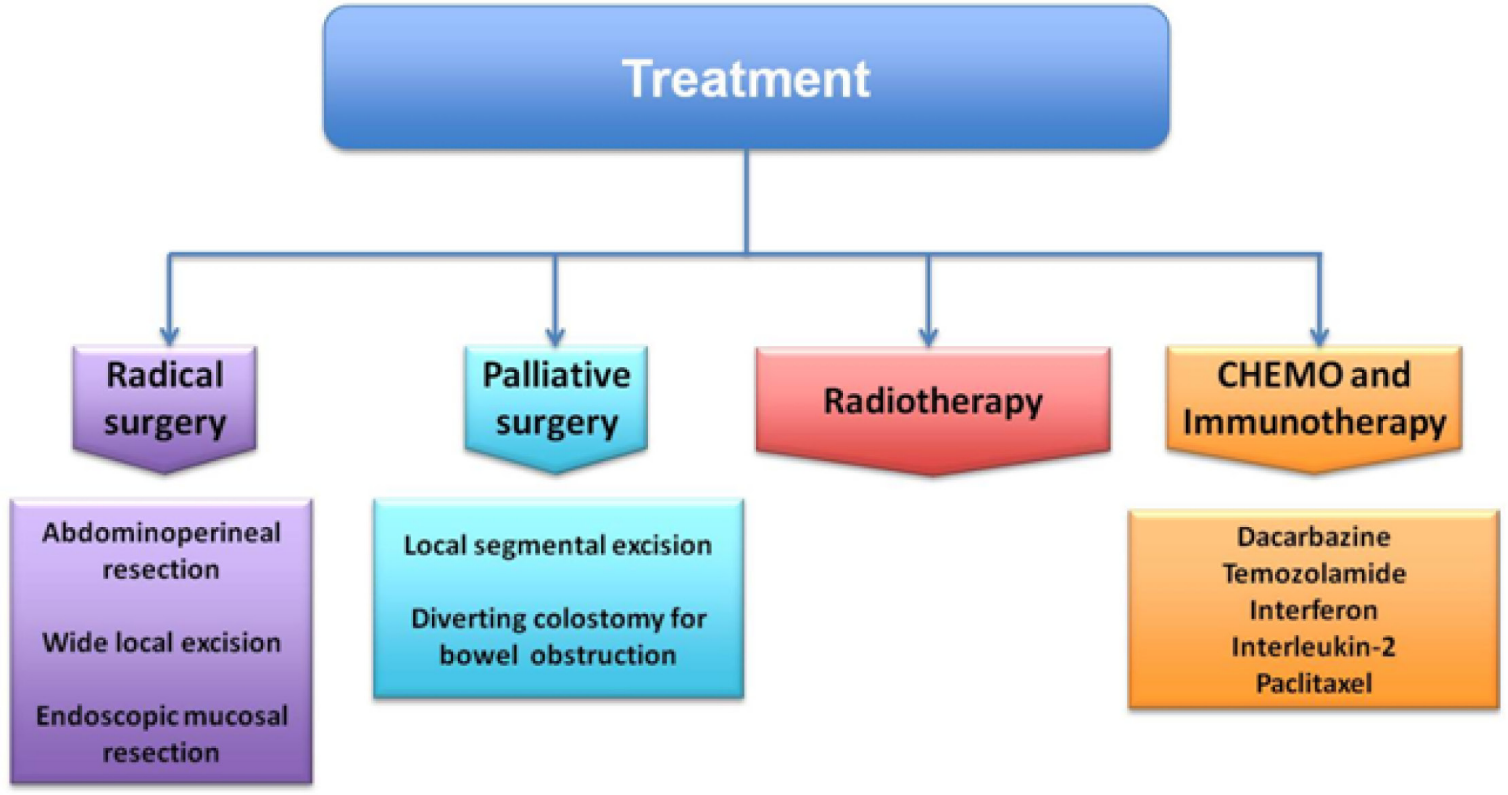
Treatment strategy of anorectal melanoma

Although traditionally APR was considered the best option treatment of loco regional disease, comparing conservative and radical procedures recent studies had reported not only no difference in survival, but also there are increasing evidences suggesting that survival outcomes may be the same between local excision and less perioperative morbidity [86–88] (Figure 2).

Palliative surgery (such as local segmental resection or a diverting colostomy for bowel obstruction) is suggested in large primary tumours or in presence of distant metastases [89].

### Medical treatment

### Adjuvant therapies

In the past decades several adjuvant therapeutic options such as immunotherapy with alfa interferon, brachytherapy with 117-Caesium and chemotherapy with dacarbazine, vincristine and nimustine hydrochloride were used [90, 91].

In mucosal melanomas, particularly in patients with positive nodal involvement, α-interferon at the dose of 20 MU/m2/day intravenously 5 day weekly for 4 weeks, followed by 10 MU/m2/day subcutaneously three times weekly for 4–8 weeks had demonstrated a significant prolongation of relapse-free-survival and overall survival [92]. Systemic side effects of interferon include haematological, autoimmune, neuropsychiatric disorders [93–96]. High dose interferon (HDI) toxicity has been raised concerns and vaccine alternatives have been studied. In terms of efficacy, Kirkwood *et al.* had found that HDI arm for 1 year showed a better response versus vaccination with GM2-gangliioside arm [97].

### Treatment in advanced and metastatic mucosal melanoma

In the management of metastatic melanoma, Interleukin-2 (IL-2), alone or in combination with chemotherapy or biotherapy, is a standard treatment. No higher long-term survival had been reported despite higher tumour response rates of combination of IL-2 with chemotherapy and/or IFN. Traditional cytotoxic chemotherapeutic agents, including decarbazine and temozolamide have been reported to be ineffective in metastatic mucosal melanoma.

Dacarbazine alone or in combination with high-dose of Interferon and Interleukin-2 is effective in 10–20% of patients with mucosal melanoma [98–100].

### Target therapies and immunotherapies

**Figure 3:**
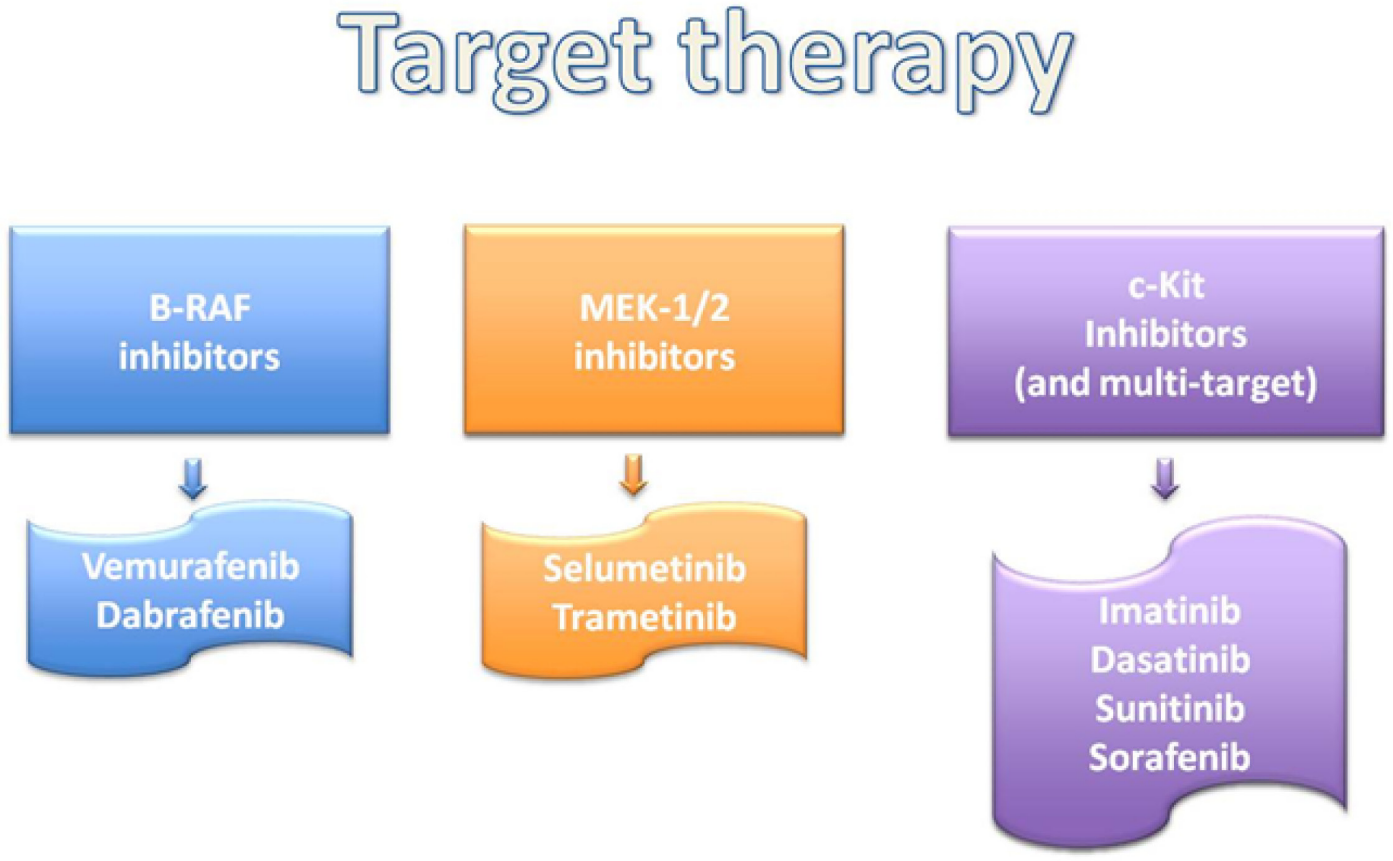
Genomic analyses based treatments

Novel immunotherapeutic strategies for metastasis are having promising results and they are developed in parallel with MAPK pathway inhibitor (Figure 2). Understanding the biology of the advanced melanoma and its immune regulation, also the treatments are evolving simultaneously. In fact, the present target in advanced melanoma treatment is focused on immune checkpoints (Figure 3).

#### Target therapies

Expression of c-Kit can be demonstrated in most melanomas by immunohistochemistry. However, studies of c-kit blockers such as imatinib have been unsuccessful [101, 102].

KIT kinase inhibitors have shown activity on most of reported mutation predicted. For instance, in presence of GIST mutation that affects the Exon 9 is necessary to double the dose of Imatinib (Figure 3).

On going phase II studies provide promising results that patient with KIT-mutated metastatic melanomas are amenable to single-agent therapy with one of the kit inhibitors imatinib, dasatinib, sunitinib or sorafenib [103–106]. Currently, they are being tested several mutated c-Kit inhibitors in the treatment of metastatic disease (imatinib, dasatinib, sunitinib or sorafenib) [107–112]. Hodi FS *et al* reported good response on treatment in a patient with KIT mutated rectal melanoma [113] and Minor DR *et al* reported a complete remission for 15 months in patients with KIT-mutated underwent to sunitinib treatment [114]. In a study a patient with mucosal melanoma that received the mTOR inhibitor everolimus constituted a partial response [115].

It seems that melanoma cells in the proximity of older fibroblast may have higher levels of scavengers of reactive oxygen species. Older fibroblast produced high amounts of secreted frizzled-related protein 2 (sFRP2), a secreted protein, which was detectable in the serum of the older mice enhanced tumour angiogenesis and lung metastasis in the BRAF V 600 E model. Elevated levels of sFRP2 reduce the ability of melanoma cells to respond to the oxidative stress. The relative scarcity of scavengers of ROS and the relative abundance of on the oxidative stress in melanoma cells, SFRP2 represent a double whammy, which, in turn, led to DNA damage [116].

Enhanced oxidative stress and DNA damage have linked not only with a more aggressive tumour phenotype, but also with resistance to BRAF target drugs, such as vemurafenib.

About 40–50% of metastatic melanomas shows activation of MAPK (mitogen-activate protein kinase) pathway [117].

Dabrafenib is classified as reversible ATP-competitive inhibithor with selectively inhibition on BRAF. In approximately 50–70% of patients with mutation in BRAF V600E or in V600K, Dabrafenib has demonstrated efficacy [118, 119]. Targeting these specific mutation, BRAF inhibitors, such as vemurafenib, dabrafenib, and encorafenib were developed. Moreover, targeting the signalling pathway of BRAF and inhibiting molecule of its downstream, such as MEK, may inactivate the MAPK pathway.

Prolongation on progression-free and overall survival had been demonstrated with a therapeutic combination of BRAF and MEK inhibitor, such as vemurafenib plus cobimetinib or dabrafenib plus Trametinib. BRAF and MAP inhibitors have shown efficacy also in BRAF V600 mutated melanoma.

Current efforts to investigate the biological and genomic characteristics of these tumours have been constrained by the low incidence of mucosal melanoma.

Mutations in ARMMs have demonstrated how melanomas are heterogeneous in their tumour biology [120]. Studying these mutations may allow finding new target molecules to develop specific treatments with better response. For instance, patients with mutation in the BRAF V 600 E gene and melanoma patients with KIT gene abberration represent two subgroup responsive respectively to the action of BRAF inhibitors and cKit blockersBRAF inhibitors, such as PCV4032 and RAF 265, induce tumour regression in up to 70% of patients with metastatic disease, whereas cKit blockers includes Imatinib, dasatinib, sunitinib and sorafenib [121]. In recent years certain patients subgroups by certain target have been described that may predict susceptibility to target therapies. However, it is currently unclear if patient will clinically benefit from target therapies that are guided by the absence or presence of susceptibility parameters [21].

One group comprises melanomas that harbour BRAF mutations. In small numbers of patients, specific BRAF inhibitors (PLX 4032 and RAF 265) induced tumour regression in up to 70% of patients with BRAF V 600 E mutated metastatic melanomas [122, 123].

Vemurafenib, a potent BRAF protein kinase inhibitor, is reserved for the treatment of metastatic melanoma associated with BRAF V 600 mutation [124]. Vemurafenib is given orally and its mechanism of action consists in the inhibition of kinase domain, decreasing cell proliferation through the phosphorylation of ERK and cyclin D1. Its activity is effective in most common mutation of BRAF V600E, but not in BRAF wt [125, 126]. Trials of Vemurafenib and Ipilimumab combination therapy are currently underway in patients with BRAF mutation.

In the absence of BRAF V 600 mutation, treatment of these advanced, refractory forms is based on the use of ipilimumab either alone or in combination with standard chemotherapy (dacarbazine).

#### Immunologic checkpoint blockade

In advanced mucosal melanoma the treatment with ipilimumab, an anti-CTLA4 antibodies, demonstrated some effectiveness with median PFS of 6.4 months and median OS 4.3 months [127].

**Figure 4:**
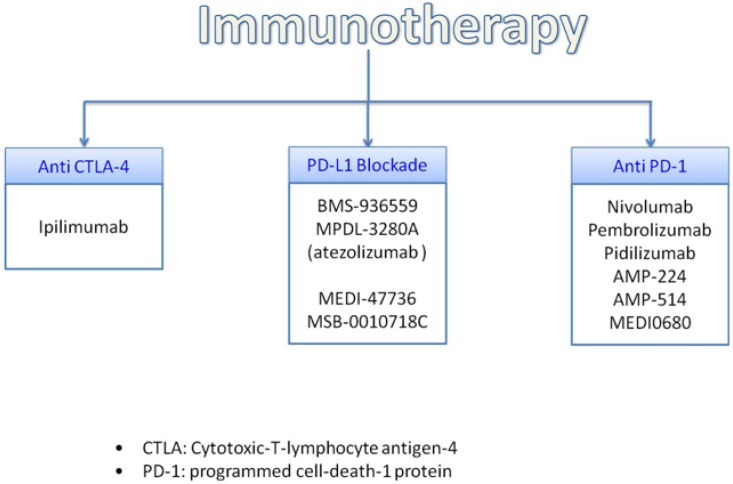
Promising immunotherapy treatment

Molecules involved in immune response that normally terminate after antigen activation represent a main target for novel therapeutic strategies (Figure 4).

#### CTLA4

Cytotoxic T- lymphocyte-associated antigen (CTLA-4 also known as CD152) is expressed on T cells, in which cells regulates the amplitude of early activation. CTLA-4 has also immunomodulating activating by downregulating cell-activation. In fact, CTLA-4 is anantagonist of B7-CD28- mediated co-stimulatory signals [128] with binding affinity to CD80/86 of 500-2500 times more than CD 28.

Since CTLA-4 acts on blockage of CD28 arrests cell cycle progression. In fact CD28 not only promotes IL-2 mRNA expression, but also is responsible of T-cell survival, T-helper cell differentiation and immunoglobulin isotype switching.

Ipilimumab is a fully human monoclonal antibody that binds CTLA4 and blocks the interaction of CTLA4, which is CD80 and CD 86 ligands; it was the first successful developed drug of the novel class of compounds named immune checkpoint inhibitors. Ipilimumab mechanism of action has an indirect effect on T-cell mediated antitumor immune responses and it had shown long-term survival of up to 20% of treated patients [129]. Ipilimumab was the first agent approved for the treatment of advanced melanoma.

#### PD1/PDl-1

Anti PD1, Anti PDL1 and PDL2 drugs have completely changed the landscape for the treatment of advanced and metastatic melanoma. Their efficacy had been showed in 20–405 of patients with proven safety with less than 10% of patients experiencing grade 3–4 adverse events. However, there are no guidelines regarding the use of PD1 and PDL1 agents. In particular, it is not established for how long these agents should be used in patients who are responding: patients, who were benefiting, were allowed to stay on treatment indefinitely.

Programmed-death (PD1) is another immune checkpoint target expressed on activated T-cells with immunosuppressive effects. Its ligands, PDL1 (B7H8) and PDL2 (B7DC) are expressed on many stromal cells, tumour cellsand other cell types. The immunosuppression of PD1 receptor is due to the interaction between T lymphocytes and tumour cells. PD1 blockage seems to be more effective towards t-cell activation than CTLA-4 inhibition.

Nivolumab (BMS-936558) and Pembrolizumab (MK-3475) are both humanized monoclonal Ig4 antigen and antagonist of PD1, which had been studied in advanced melanoma and other solid tumours, whereas other PD-1 and PDL1 inhibitors are still under evaluation.

Le Min reported one case of anti-PD1 treatment in a patient with advanced mucosal melanoma with a durable near-complete response [130, 131].

### Angiogenesis targeted therapies

Melanoma is a highly angiogenic tumor. Angiogenesis represents an important process to modulate in melanoma, as proangiogenic ligands and their receptors are overexpressed and have been found to correlate with disease progression and prognosis [132] (Figure 1). The antiangiogenetic treatment has been proved to be a potential strategy.

A growing clinical trial has incorporated bevacizumab combined with nab-paclitaxel in advanced and metastatic melanoma [133].

Another phase II trial (Endostatin plus dacarbazine) showed in 16 cases of mucosal melanomas a significant improvement in progression free survival and in overall survival [134]. Endostatin is an endogenous angiogenesis inhibitor and a tumor suppressor [135].

### Prognosis

The anorectal melanoma is associated with extremely poor prognosis (Table 1) with all surgical approaches achieving a 5-year survival rate of less than 20%. Long term survival has been demonstrated in patients who have undergone abdominoperineal resection with extended lymph node dissection, including patients with lymphatic metastases; thus abdomino-perineal resection has been widely recommended.

**Table 1:** Prognosis of mucosal melanomas [Modified from https://www.dermnetnz.org/topics/mucosal-melanoma]

Type	5-year survival rate
Head and neck melanomas	12–30%
Vulvar melanomas	24–77%
Vaginal melanomas	5–25%
Anorectal melanomas	20%

In western countries, there is no significant difference in survival rate between rectal resection with a sufficient safety margin and local excision and most surgical treatment is considered palliative surgery therapy due to the possibility of disease already being advanced at the time of onset and diagnosis.

The small numbers of ARMM reports make difficult any meaningful analysis for prognostic factor such as stage at diagnosis, lymph node status and tumour thickness [136].

The use of colonscopy and biopsy can establish a correct diagnosis. The poor survival in ARMM may be explained by the tendency to late diagnosis. Prognosis depends on the depth of invasion, duration of symptoms, inguinal lymph node involvement, presence of distant metastasis, tumor stage and the presence of amelanotic melanoma (Table 2) [137].

**Table 2:** Prognistic factors of anorectal melanoma

Prognistic factors of anorectal melanoma
Size
Dept of invasion
Mode of treatment
Duration of symptoms
Stage of the disease
Nodal involvement
Molecular markers like PCNA and Ki-67

## CONCLUSIONS

Little is known about the prognostic parameters, staging and treatment protocols [138] The anorectal melanoma is rare associated with worse prognosis due to the late diagnosis.

This tumour is characterized by aggressive behaviour, poor outcome as result of late diagnosis and fast tumour growth in the rich vascular and lymphatic supply of the ano-rectal mucosa [139].

Considerable effort has been devoted to the identification of molecular determinants underlying melanoma chemo and immunoresistence [140]. Today anorectal melanoma has clinical, genetic and biologic similarities to other mucosal melanomas. Anorectal melanomas are a subtype of mucosal melanoma and mucosal melanoma are a subtype of melanoma in general. Most manage of anorectal melanoma and melanoma mucosal has translated of cutaneous melanoma [140].

The biologic and clinical heterogeneity of cutaneous malignancies and mucosal melanomas provide a number of unique opportunities and challenges for preclinic and clinical studies.

New targeted therapies (and immunotherapies) are under investigation for patients with both metastatic and primitive melanoma.

Better understanding of genetic aberrations in mucosal melanoma could provide development of targeting and effective treatment [141–143].

## References

[1] Longo WE, Vernava AM, Wade TP, Coplin MA, Virgo KS, Johnson FE (1995). Rare anal canal cancers in the U.S. veteran: patterns of disease and results of treatment. Am Surg.

[2] Hillenbrand A, Barth TF, Henne-Bruns D, Formentini A (2008). Anorectal amelanotic melanoma. Colorectal Dis.

[3] Weyandt GH, Eggert AO, Houf M, Raulf F, Bröcker EB, Becker JC (2003). Anorectal melanoma: surgical management guidelines according to tumour thickness. Br J Cancer.

[4] Malaguarnera M, Giordano M, Rando A, Puzzo L, Trainiti M, Consoli AS, Catania VE (2011). Intestinal lymphoma: a case report. Eur Rev Med Pharmacol Sci.

[5] Nakhleh RE, Wick MR, Rocamora A, Swanson PE, Dehner LP (1990). Morphologic diversity in malignant melanomas. Am J Clin Pathol.

[6] Fenger C, Frisch M, Jass JJ, Williams GT, Hilden J (2000). Anal cancer subtype reproducibility study. Virchows Arch.

[7] Balachandra B, Marcus V, Jass JR (2007). Poorly differentiated tumours of the anal canal: a diagnostic strategy for the surgical pathologist. Histopathology.

[8] Coté TR, Sobin LH (2009). Primary melanomas of the esophagus and anorectum: epidemiologic comparison with melanoma of the skin. Melanoma Res.

[9] Jemal A, Siegel R, Ward E, Hao Y, Xu J, Murray T, Thun MJ (2008). Cancer statistics, 2008. CA Cancer J Clin.

[10] Weinstock MA (1993). Epidemiology and prognosis of anorectal melanoma. Gastroenterology.

[11] Zhang S, Gao F, Wan D (2010). Effect of misdiagnosis on the prognosis of anorectal malignant melanoma. J Cancer Res Clin Oncol.

[12] Felz MW, Winburn GB, Kallab AM, Lee JR (2001). Anal melanoma: an aggressive malignancy masquerading as hemorrhoids. South Med J.

[13] Belli F, Gallino GF, Lo Vullo S, Mariani L, Poiasina E, Leo E (2009). Melanoma of the anorectal region: the experience of the National Cancer Institute of Milano. Eur J Surg Oncol.

[14] Iddings DM, Fleisig AJ, Chen SL, Faries MB, Morton DL (2010). Practice patterns and outcomes for anorectal melanoma in the USA, reviewing three decades of treatment: is more extensive surgical resection beneficial in all patients?. Ann Surg Oncol.

[15] Adler MJ, White CR (1997). Amelanotic malignant melanoma. Semin Cutan Med Surg.

[16] Gray-Schopfer V, Wellbrock C, Marais R (2007). Melanoma biology and new targeted therapy. Nature.

[17] Pizzichetta MA, Talamini R, Stanganelli I, Puddu P, Bono R, Argenziano G, Veronesi A, Trevisan G, Rabinovitz H, Soyer HP (2004). Amelanotic/hypomelanotic melanoma: clinical and dermoscopic features. Br J Dermatol.

[18] Pessaux P, Pocard M, Elias D, Duvillard P, Avril MF, Zimmerman P, Lasser P (2004). Surgical management of primary anorectal melanoma. Br J Surg.

[19] Trzcinski R, Kujawski R, Mik M, Sygut A, Dziki L, Dziki A (2010). Malignant melanoma of the anorectum—a rare entity. Langenbecks Arch Surg.

[20] Di Rosa M, Malaguarnera G, De Gregorio C, Drago F, Malaguarnera L (2013). Evaluation of CHI3L-1 and CHIT-1 expression in differentiated and polarized macrophages. Inflammation.

[21] Mikkelsen LH, Larsen AC, von Buchwald C, Drzewiecki KT, Prause JU, Heegaard S (2016). Mucosal malignant melanoma - a clinical, oncological, pathological and genetic survey. APMIS.

[22] Chisari CG, Stagni E, Di Mauro M, Di Mauro M, Giordano M, Fichera SS, Motta M, Chisari EM, Chisari G (2014). Risk factors for ocular surface disorders in patients with type 2 diabetes. Acta Med Mediter.

[23] Tollenson WH (2005). Human melanocyte biology, toxicology, and pathology. J Environ Sci Health C Environ Carcinog Ecotoxicol Rev.

[24] Mackintosh JA (2001). The antimicrobial properties of melanocytes, melanosomes and melanin and the evolution of black skin. J Theor Biol.

[25] Plonka PM, Passeron T, Brenner M, Tobin DJ, Shibahara S, Thomas A, Slominski A, Kadekaro AL, Hershkovitz D, Peters E, Nordlund JJ, Abdel-Malek Z, Takeda K (2009). What are melanocytes really doing all day long..?. Exp Dermatol.

[26] Malaguarnera G, Giordano M, Nunnari G, Bertino G, Malaguarnera M (2014). Gut microbiota in alcoholic liver disease: pathogenetic role and therapeutic perspectives. World J Gastroenterol.

[27] Chisari G, Rampello L, Chisari EM, Catania VE, Greco C, Stagno E, Chisari CG (2016). Microbiology synergism between tear substitutes and symbiotic treatment of patients with irritable bowel syndrome. Acta Med Mediter.

[28] Shain AH, Bastian BC (2016). From melanocytes to melanomas. Nat Rev Cancer.

[29] Takeda K, Takahashi NH, Shibahara S (2007). Neuroendocrine functions of melanocytes: beyond the skin-deep melanin maker. Tohoku J Exp Med.

[30] Marks MS, Seabra MC (2001). The melanosome: membrane dynamics in black and white. Nat Rev Mol Cell Biol.

[31] Feller L, Masilana A, Khammissa RA, Altini M, Jadwat Y, Lemmer J (2014). Melanin: the biophysiology of oral melanocytes and physiological oral pigmentation. Head Face Med.

[32] Chi A, Valencia JC, Hu ZZ, Watabe H, Yamaguchi H, Mangini NJ, Huang H, Canfield VA, Cheng KC, Yang F, Abe R, Yamagishi S, Shabanowitz J (2006). Proteomic and bioinformatic characterization of the biogenesis and function of melanosomes. J Proteome Res.

[33] Wong CW, Fan YS, Chan TL, Chan AS, Ho LC, Ma TK, Yuen ST, Leung SY, Cancer Genome Project (2005). BRAF and NRAS mutations are uncommon in melanomas arising in diverse internal organs. J Clin Pathol.

[34] Timmers TK, Schadd EM, Monkelbaan JF, Meij V (2013). Survival after resection of a primary malignant melanoma of the small intestine in a young patient: report of a case. Case Rep Gastroenterol.

[35] Sachs DL, Lowe L, Chang AE, Carson E, Johnson TM (1999). Do primary small intestinal melanomas exist? Report of a case. J Am Acad Dermatol.

[36] Blecker D, Abraham S, Furth EE, Kochman ML (1999). Melanoma in the gastrointestinal tract. Am J Gastroenterol.

[37] Wang S, Sun S, Liu X, Ge N, Wang G, Guo J, Liu W, Wang S (2017). Endoscopic diagnosis of primary anorectal melanoma. Oncotarget.

[38] Latteri S, Malaguarnera G, Mannino M, Pesce A, Currò G, Tamburrini S, Scuderi M (2017). Ultrasound as point of care in management of polytrauma and its complication. J Ultrasound.

[39] Helmke BM, Otto HF (2004). [Anorectal melanoma. A rare and highly malignant tumor entity of the anal canal]. [Article in German]. Pathologe.

[40] Flaherty KT, Hodi FS, Fisher DE (2012). From genes to drugs: targeted strategies for melanoma. Nat Rev Cancer.

[41] La Greca G, Santangelo A, Primo S, Sofia M, Latteri S, Russello D, Magro G (2014). Clinical and diagnostic problems of desmoid-type fibromatosis of the mesentery: case report and review of the literature. Ann Ital Chir.

[42] Catania V, Consoli A, Cavallaro A, Liardo RL, Malaguarnera M (2010). The neo-adjuvant treatment in gastrointestinal stromal tumor. Eur Rev Med Pharmacol Sci.

[43] Catania VE, Vecchio M, Malaguarnera M, Madeddu R, Malaguarnera G, Latteri S (2017). Tumor lysis syndrome in an extraskeletal osteosarcoma: a case report and review of the literature. J Med Case Reports.

[44] Beadling C, Jacobson-Dunlop E, Hodi FS, Le C, Warrick A, Patterson J, Town A, Harlow A, Cruz F, Azar S, Rubin BP, Muller S, West R (2008). KIT gene mutations and copy number in melanoma subtypes. Clin Cancer Res.

[45] Santi R, Simi L, Fucci R, Paglierani M, Pepi M, Pinzani P, Merelli B, Santucci M, Botti G, Urso C, Massi D (2015). KIT genetic alterations in anorectal melanomas. J Clin Pathol.

[46] Di Rosa M, De Gregorio C, Malaguarnera G, Tuttobene M, Biazzo F, Malaguarnera L (2013). Evaluation of AMCase and CHIT-1 expression in monocyte macrophages lineage. Mol Cell Biochem.

[47] Hintzsche JD, Gorden NT, Amato CM, Kim J, Wuensch KE, Robinson SE, Applegate AJ, Couts KL, Medina TM, Wells KR, Wisell JA, McCarter MD, Box NF (2017). Whole-exome sequencing identifies recurrent SF3B1 R625 mutation and comutation of NF1 and KIT in mucosal melanoma. Melanoma Res.

[48] Sullivan RJ, Lorusso PM, Flaherty KT (2013). The intersection of immune-directed and molecularly targeted therapy in advanced melanoma: where we have been, are, and will be. Clin Cancer Res.

[49] Sullivan RJ, Flaherty K (2013). MAP kinase signaling and inhibition in melanoma. Oncogene.

[50] Maldonado JL, Fridlyand J, Patel H, Jain AN, Busam K, Kageshita T, Ono T, Albertson DG, Pinkel D, Bastian BC (2003). Determinants of BRAF mutations in primary melanomas. J Natl Cancer Inst.

[51] Cohen Y, Rosenbaum E, Begum S, Goldenberg D, Esche C, Lavie O, Sidransky D, Westra WH (2004). Exon 15 BRAF mutations are uncommon in melanomas arising in nonsun-exposed sites. Clin Cancer Res.

[52] Edwards RH, Ward MR, Wu H, Medina CA, Brose MS, Volpe P, Nussen-Lee S, Haupt HM, Martin AM, Herlyn M, Lessin SR, Weber BL (2004). Absence of BRAF mutations in UV-protected mucosal melanomas. J Med Genet.

[53] Kruijff S, Bastiaannet E, Kobold AC, van Ginkel RJ, Suurmeijer AJ, Hoekstra HJ (2009). S-100B concentrations predict disease-free survival in stage III melanoma patients. Ann Surg Oncol.

[54] Kluger HM, Hoyt K, Bacchiocchi A, Mayer T, Kirsch J, Kluger Y, Sznol M, Ariyan S, Molinaro A, Halaban R (2011). Plasma markers for identifying patients with metastatic melanoma. Clin Cancer Res.

[55] Miliotes G, Lyman GH, Cruse CW, Puleo C, Albertini PA, Rapaport D, Glass F, Fenske N, Soriano T, Cuny C, Van Voorhis N, Reintgen D (1996). Evaluation of new putative tumor markers for melanoma. Ann Surg Oncol.

[56] Bosserhoff AK, Kaufmann M, Kaluza B, Bartke I, Zirngibl H, Hein R, Stolz W, Buettner R (1997). Melanoma-inhibiting activity, a novel serum marker for progression of malignant melanoma. Cancer Res.

[57] Schmidt H, Johansen JS, Gehl J, Geertsen PF, Fode K, von der Maase H (2006). Elevated serum level of YKL-40 is an independent prognostic factor for poor survival in patients with metastatic melanoma. Cancer.

[58] Perrotta R, Bevelacqua Y, Malaguarnera G, Paladina I, Giordano M, Malaguarnera M (2010). Serum markers of cutaneous melanoma. Front Biosci (Elite Ed).

[59] Cabrera-Pastor A, Malaguarnera M, Taoro-Gonzalez L, Llansola M, Felipo V (2016). Extracellular cGMP Modulates Learning Biphasically by Modulating Glycine Receptors, CaMKII and Glutamate-Nitric Oxide-cGMP Pathway. Sci Rep.

[60] Smit LH, Nieweg OE, Korse CM, Bonfrer JM, Kroon BB (2005). Significance of serum S-100B in melanoma patients before and after sentinel node biopsy. J Surg Oncol.

[61] Latteri S, Teodoro M, Malaguarnera M, Mannino M, Currò G, La Greca G (2017). Abdominal perineal resection or wilde local excision in primary anorectal malignant melanoma. Case report and review. Ann Med Surg (Lond).

[62] Ghanem G, Loir B, Morandini R, Sales F, Lienard D, Eggermont A, Lejeune F, EORTC Melanoma Group (2001). On the release and half-life of S100B protein in the peripheral blood of melanoma patients. Int J Cancer.

[63] Bosserhoff AK, Golob M, Buettner R, Landthaler M, Hein R (1998). [MIA (“melanoma inhibitory activity”). Biological functions and clinical relevance in malignant melanoma]. [Article in German]. Hautarzt.

[64] Koehler MR, Bosserhoff A, von Beust G, Bauer A, Blesch A, Buettner R, Schlegel J, Bogdahn U, Schmid M (1996). Assignment of the human melanoma inhibitory activity gene (MIA) to 19q13.32-q13.33 by fluorescence *in situ* hybridization (FISH). Genomics.

[65] Buzaid AC, Sandler AB, Hayden CL, Scinto J, Poo WJ, Clark MB, Hotchkiss S (1994). Neuron-specific enolase as a tumor marker in metastatic melanoma. Am J Clin Oncol.

[66] Pennisi M, Bertino G, Gagliano C, Malaguarnera M, Bella R, Borzì AM, Madeddu R, Drago F, Malaguarnera G (2017). Resveratrol in Hepatitis C Patients Treated with Pegylated-Interferon-α-2b and Ribavirin Reduces Sleep Disturbance. Nutrients.

[67] Johansen JS (2006). Studies on serum YKL-40 as a biomarker in diseases with inflammation, tissue remodelling, fibroses and cancer. Dan Med Bull.

[68] Johansen JS, Jensen BV, Roslind A, Nielsen D, Price PA (2006). Serum YKL-40, a new prognostic biomarker in cancer patients?. Cancer Epidemiol Biomarkers Prev.

[69] Garbe C, Leiter U, Ellwanger U, Blaheta HJ, Meier F, Rassner G, Schittek B (2003). Diagnostic value and prognostic significance of protein S-100beta, melanoma-inhibitory activity, and tyrosinase/MART-1 reverse transcription-polymerase chain reaction in the follow-up of high-risk melanoma patients. Cancer.

[70] Shao R, Hamel K, Petersen L, Cao QJ, Arenas RB, Bigelow C, Bentley B, Yan W (2009). YKL-40, a secreted glycoprotein, promotes tumor angiogenesis. Oncogene.

[71] Riabov V, Gudima A, Wang N, Mickley A, Orekhov A, Kzhyshkowska J (2014). Role of tumor associated macrophages in tumor angiogenesis and lymphangiogenesis. Front Physiol.

[72] Di Rosa M, Malaguarnera G, De Gregorio C, D’Amico F, Mazzarino MC, Malaguarnera L (2013). Modulation of chitotriosidase during macrophage differentiation. Cell Biochem Biophys.

[73] Krogh M, Christensen I, Bouwhuis M, Johansen JS, Nørgaard P, Schmidt H, Hansson J, Suciu S, Eggermont AM, Bastholt L, Nordic Melanoma Group and EORTC Melanoma Group (2016). Prognostic and predictive value of YKL-40 in stage IIB-III melanoma. Melanoma Res.

[74] Woodruff WW, Djang WT, McLendon RE, Heinz ER, Voorhees DR (1987). Intracerebral malignant melanoma: high-field-strength MR imaging. Radiology.

[75] Winkler N, Rezvani M, Heilbrun M, Shaaban A (2013). Utility of dual phase liver CT for metastatic melanoma staging and surveillance. Eur J Radiol.

[76] Matsuoka H, Nakamura A, Iwamoto K, Sugiyama M, Hachiya J, Atomi Y, Masaki T (2005). Anorectal malignant melanoma: preoperative usefulness of magnetic resonance imaging. J Gastroenterol.

[77] Barbagallo F, Latteri S, Sofia M, Ricotta A, Castello G, Chisari A, Randazzo V, La Greca G (2010). Appendicular tuberculosis: the resurgence of an old disease with difficult diagnosis. World J Gastroenterol.

[78] Singer M, Mutch MG (2006). Anal melanoma. Clin Colon Rectal Surg.

[79] Prasad ML, Jungbluth AA, Patel SG, Iversen K, Hoshaw-Woodard S, Busam KJ (2004). Expression and significance of cancer testis antigens in primary mucosal melanoma of the head and neck. Head Neck.

[80] Antoniuk PM, Tjandra JJ, Webb BW, Petras RE, Milsom JW, Fazio VW (1993). Anorectal malignant melanoma has a poor prognosis. Int J Colorectal Dis.

[81] Tanaka S, Ohta T, Fujimoto T, Makino Y, Murakami I (2008). Endoscopic mucosal resection of primary anorectal malignant melanoma: a case report. Acta Med Okayama.

[82] Cacopardo B, Pinzone M, Berretta S, Fisichella R, Di Vita M, Zanghì G, Cappellani A, Nunnari G, Zanghì A (2013). Localized and systemic bacterial infections in necrotizing pancreatitis submitted to surgical necrosectomy or percutaneous drainage of necrotic secretions. BMC Surg.

[83] Slingluff CL, Vollmer RT, Seigler HF (1990). Anorectal melanoma: clinical characteristics and results of surgical management in twenty-four patients. Surgery.

[84] Buissin D, Sterle A, Schmiegelow P, Wassenberg D, Ambe PC (2015). Primary anorectal malignant melanoma: a rare but aggressive tumor: report of a case. World J Surg Oncol.

[85] Bullard KM, Tuttle TM, Rothenberger DA, Madoff RD, Baxter NN, Finne CO, Spencer MP (2003). Surgical therapy for anorectal melanoma. J Am Coll Surg.

[86] Kiran RP, Rottoli M, Pokala N, Fazio VW (2010). Long-term outcomes after local excision and radical surgery for anal melanoma: data from a population database. Dis Colon Rectum.

[87] Yeh JJ, Shia J, Hwu WJ, Busam KJ, Paty PB, Guillem JG, Coit DG, Wong WD, Weiser MR (2006). The role of abdominoperineal resection as surgical therapy for anorectal melanoma. Ann Surg.

[88] Zhou HT, Zhou ZX, Zhang HZ, Bi JJ, Zhao P (2010). Wide local excision could be considered as the initial treatment of primary anorectal malignant melanoma. Chin Med J (Engl).

[89] Koch SE, Lange JR (2000). Amelanotic melanoma: the great masquerader. J Am Acad Dermatol.

[90] Moozar KL, Wong CS, Couture J (2003). Anorectal malignant melanoma: treatment with surgery or radiation therapy, or both. Can J Surg.

[91] Grob JJ, Dreno B, de la Salmonière P, Delaunay M, Cupissol D, Guillot B, Souteyrand P, Sassolas B, Cesarini JP, Lionnet S, Lok C, Chastang C, Bonerandi JJ, French Cooperative Group on Melanoma (1998). Randomised trial of interferon alpha-2a as adjuvant therapy in resected primary melanoma thicker than 1.5 mm without clinically detectable node metastases. Lancet.

[92] Kirkwood JM, Strawderman MH, Ernstoff MS, Smith TJ, Borden EC, Blum RH (1996). Interferon alfa-2b adjuvant therapy of high-risk resected cutaneous melanoma: the Eastern Cooperative Oncology Group Trial EST 1684. J Clin Oncol.

[93] Malaguarnera G, Bertino G, Chisari G, Motta M, Vecchio M, Vacante M, Caraci F, Greco C, Drago F, Nunnari G, Malaguarnera M (2016). Silybin supplementation during HCV therapy with pegylated interferon-α plus ribavirin reduces depression and anxiety and increases work ability. BMC Psychiatry.

[94] Das G, Gupta S, Shukla PJ, Jagannath P (2003). Anorectal melanoma: a large clinicopathologic study from India. Int Surg.

[95] Malaguarnera M, Vacante M, Russo C, Gargante MP, Giordano M, Bertino G, Neri S, Malaguarnera M, Galvano F, Li Volti G (2011). Rosuvastatin reduces nonalcoholic fatty liver disease in patients with chronic hepatitis C treated with α-interferon and ribavirin: rosuvastatin reduces NAFLD in HCV patients. Hepat Mon.

[96] Malaguarnera M, Vacante M, Giordano M, Motta M, Bertino G, Pennisi M, Neri S, Malaguarnera M, Li Volti G, Galvano F (2011). L-carnitine supplementation improves hematological pattern in patients affected by HCV treated with Peg interferon-α 2b plus ribavirin. World J Gastroenterol.

[97] Kirkwood JM, Farkas DL, Chakraborty A, Dyer KF, Tweardy DJ, Abernethy JL, Edington HD, Donnelly SS, Becker D (1999). Systemic interferon-alpha (IFN-alpha) treatment leads to Stat3 inactivation in melanoma precursor lesions. Mol Med.

[98] Gavriel H, McArthur G, Sizeland A, Henderson M (2011). Review: mucosal melanoma of the head and neck. Melanoma Res.

[99] Wang X, Si L, Guo J (2014). Treatment algorithm of metastatic mucosal melanoma. Chin Clin Oncol.

[100] Komatsubara KM, Jeter J, Carvajal RD, Margolin K, Schadendorf D, Hauschild A (2017). Advances in the Treatment of Advanced Extracutaneous Melanomas and Nonmelanoma Skin Cancers. Am Soc Clin Oncol Educ Book.

[101] Kim KB, Eton O, Davis DW, Frazier ML, McConkey DJ, Diwan AH, Papadopoulos NE, Bedikian AY, Camacho LH, Ross MI, Cormier JN, Gershenwald JE, Lee JE (2008). Phase II trial of imatinib mesylate in patients with metastatic melanoma. Br J Cancer.

[102] Ugurel S, Hildenbrand R, Zimpfer A, La Rosée P, Paschka P, Sucker A, Keikavoussi P, Becker JC, Rittgen W, Hochhaus A, Schadendorf D (2005). Lack of clinical efficacy of imatinib in metastatic melanoma. Br J Cancer.

[103] Zhu Y, Si L, Kong Y, Chi Z, Yuan X, Cui C, Sheng X, Guo J, Shen L (2009). Response to sunitinib in Chinese KIT-mutated metastatic mucosal melanoma. J Clin Oncol (Meeting Abstracts).

[104] Perfetti V, Laurini E, Aulić S, Fermeglia M, Riboni R, Lucioni M, Dallera E, Delfanti S, Pugliese L, Latteri FS, Pietrabissa A, Pricl S (2017). Molecular and functional characterization of a new 3′ end KIT juxtamembrane deletion in a duodenal GIST treated with neoadjuvant Imatinib. Oncotarget.

[105] Malaguarnera G, Latteri S, Catania VE, Malaguarnera M (2017). Reduction of cardiovascular risk in subjects with high lipoprotein (a) levels. J Thorac Dis.

[106] Lutzky J (2010). New therapeutic options in the medical management of advanced melanoma. Semin Cutan Med Surg.

[107] Lutzky J, Bauer J, Bastian BC (2008). Dose-dependent, complete response to imatinib of a metastatic mucosal melanoma with a K642E KIT mutation. Pigment Cell Melanoma Res.

[108] Hodi FS, Friedlander P, Corless CL, Heinrich MC, Mac Rae S, Kruse A, Jagannathan J, Van den Abbeele AD, Velazquez EF, Demetri GD, Fisher DE (2008). Major response to imatinib mesylate in KIT-mutated melanoma. J Clin Oncol.

[109] Satzger I, Küttler U, Völker B, Schenck F, Kapp A, Gutzmer R (2010). Anal mucosal melanoma with KIT-activating mutation and response to imatinib therapy—case report and review of the literature. Dermatology.

[110] Kluger HM, Dudek A, McCann C, Rink L, Ritacco J, Adrada C, Phouyaphone N, Southard N, Sznol M (2009). A phase II trial of dasatinib in advanced melanoma. J Clin Oncol.

[111] Minor DR, Kashani-Sabet M, Garrido M, O’Day SJ, Hamid O, Bastian BC (2012). Sunitinib therapy for melanoma patients with KIT mutations. Clin Cancer Res.

[112] Quintás-Cardama A, Lazar AJ, Woodman SE, Kim K, Ross M, Hwu P (2008). Complete response of stage IV anal mucosal melanoma expressing KIT Val560Asp to the multikinase inhibitor sorafenib. Nat Clin Pract Oncol.

[113] Malaguarnera G, Catania VE, Francaviglia A, Malaguarnera M, Drago F, Motta M, Latteri S (2017). Lipoprotein(a) in patients with hepatocellular carcinoma and portal vein thrombosis. Aging Clin Exp Res.

[114] Asara Y, Melis A, De Luca LM, Bozzo C, Castiglia P, Chessa G, Piras P, Karligkiotis A, Bandiera P, Malaguarnera M, Marchal JA, Madeddu R (2016). Influence of metals on rhinosinusal polyposis in Sardinian population (Italy). Environ Sci Pollut Res Int.

[115] Ross M, Pezzi C, Pezzi T, Meurer D, Hickey R, Balch C (1990). Patterns of failure in anorectal melanoma. A guide to surgical therapy. Arch Surg.

[116] Kaur A, Webster MR, Marchbank K, Behera R, Ndoye A, Kugel CH, Dang VM, Appleton J, O’Connell MP, Cheng P, Valiga AA, Morissette R, McDonnell NB (2016). Corrigendum: sFRP2 in the aged microenvironment drives melanoma metastasis and therapy resistance. Nature.

[117] Eggermont AM, Spatz A, Robert C (2014). Cutaneous melanoma. Lancet.

[118] Long GV, Trefzer U, Davies MA, Kefford RF, Ascierto PA, Chapman PB, Puzanov I, Hauschild A, Robert C, Algazi A, Mortier L, Tawbi H, Wilhelm T (2012). Dabrafenib in patients with Val600Glu or Val600Lys BRAF-mutant melanoma metastatic to the brain (BREAK-MB): a multicentre, open-label, phase 2 trial. Lancet Oncol.

[119] Ascierto PA, Minor D, Ribas A, Lebbe C, O’Hagan A, Arya N, Guckert M, Schadendorf D, Kefford RF, Grob JJ, Hamid O, Amaravadi R, Simeone E (2013). Phase II trial (BREAK-2) of the BRAF inhibitor dabrafenib (GSK2118436) in patients with metastatic melanoma. J Clin Oncol.

[120] Satzger I, Schaefer T, Kuettler U, Broecker V, Voelker B, Ostertag H, Kapp A, Gutzmer R (2008). Analysis of c-KIT expression and KIT gene mutation in human mucosal melanomas. Br J Cancer.

[121] Batus M, Waheed S, Ruby C, Petersen L, Bines SD, Kaufman HL (2013). Optimal management of metastatic melanoma: current strategies and future directions. Am J Clin Dermatol.

[122] Hersey P, Bastholt L, Chiarion-Sileni V, Cinat G, Dummer R, Eggermont AM, Espinosa E, Hauschild A, Quirt I, Robert C, Schadendorf D (2009). Small molecules and targeted therapies in distant metastatic disease. Ann Oncol.

[123] Flaherty KT, Smalley KS (2009). Preclinical and clinical development of targeted therapy in melanoma: attention to schedule. Pigment Cell Melanoma Res.

[124] Flaherty KT, Puzanov I, Kim KB, Ribas A, McArthur GA, Sosman JA, O’Dwyer PJ, Lee RJ, Grippo JF, Nolop K, Chapman PB (2010). Inhibition of mutated, activated BRAF in metastatic melanoma. N Engl J Med.

[125] Chapman PB, Hauschild A, Robert C, Haanen JB, Ascierto P, Larkin J, Dummer R, Garbe C, Testori A, Maio M, Hogg D, Lorigan P, Lebbe C, BRIM-3 Study Group (2011). Improved survival with vemurafenib in melanoma with BRAF V600E mutation. N Engl J Med.

[126] Bollag G, Hirth P, Tsai J, Zhang J, Ibrahim PN, Cho H, Spevak W, Zhang C, Zhang Y, Habets G, Burton EA, Wong B, Tsang G (2010). Clinical efficacy of a RAF inhibitor needs broad target blockade in BRAF-mutant melanoma. Nature.

[127] Del Vecchio M, Di Guardo L, Ascierto PA, Grimaldi AM, Sileni VC, Pigozzo J, Ferraresi V, Nuzzo C, Rinaldi G, Testori A, Ferrucci PF, Marchetti P, De Galitiis F (2014). Efficacy and safety of ipilimumab 3mg/kg in patients with pretreated, metastatic, mucosal melanoma. Eur J Cancer.

[128] Orabona C, Grohmann U, Belladonna ML, Fallarino F, Vacca C, Bianchi R, Bozza S, Volpi C, Salomon BL, Fioretti MC, Romani L, Puccetti P (2004). CD28 induces immunostimulatory signals in dendritic cells via CD80 and CD86. Nat Immunol.

[129] Wolchok JD, Neyns B, Linette G, Negrier S, Lutzky J, Thomas L, Waterfield W, Schadendorf D, Smylie M, Guthrie T, Grob JJ, Chesney J, Chin K (2010). Ipilimumab monotherapy in patients with pretreated advanced melanoma: a randomised, double-blind, multicentre, phase 2, dose-ranging study. Lancet Oncol.

[130] Min L, Hodi FS (2014). Anti-PD1 following ipilimumab for mucosal melanoma: durable tumor response associated with severe hypothyroidism and rhabdomyolysis. Cancer Immunol Res.

[131] Thierauf J, Veit JA, Hess J, Treiber N, Lisson C, Weissinger SE, Bommer M, Hoffmann TK (2017). Checkpoint inhibition for advanced mucosal melanoma. Eur J Dermatol.

[132] La Greca G, Pesce A, Vitale M, Mannino M, Di Marco F, Di Blasi M, Lombardo R, Puleo S, Russello D, Latteri S (2017). Efficacy of the Laparoendoscopic “Rendezvous” to Treat Cholecystocholedocholithiasis in 210 Consecutive Patients: A Single Center Experience. Surg Laparosc Endosc Percutan Tech.

[133] Boasberg P, Cruickshank S, Hamid O, O'Day S, Weber R, Spitler L (2009). Nab-paclitaxel and bevacizumab as first-line therapy in patients with unresectable stage III and IV melanoma. J Clin Oncol.

[134] Cui C, Mao L, Chi Z, Si L, Sheng X, Kong Y, Li S, Lian B, Gu K, Tao M, Song X, Lin T, Ren X (2013). A phase II, randomized, double-blind, placebo-controlled multicenter trial of Endostar in patients with metastatic melanoma. Mol Ther.

[135] Sund M, Hamano Y, Sugimoto H, Sudhakar A, Soubasakos M, Yerramalla U, Benjamin LE, Lawler J, Kieran M, Shah A, Kalluri R (2005). Function of endogenous inhibitors of angiogenesis as endothelium-specific tumor suppressors. Proc Natl Acad Sci USA.

[136] Brady MS, Kavolius JP, Quan SH (1995). Anorectal melanoma. A 64-year experience at Memorial Sloan-Kettering Cancer Center. Dis Colon Rectum.

[137] Row D, Weiser MR (2009). Anorectal Melanoma. Clin Colon Rectal Surg.

[138] Balch CM, Gershenwald JE, Soong SJ, Thompson JF, Atkins MB, Byrd DR, Buzaid AC, Cochran AJ, Coit DG, Ding S, Eggermont AM, Flaherty KT, Gimotty PA (2009). Final version of 2009 AJCC melanoma staging and classification. J Clin Oncol.

[139] Meguerditchian AN, Meterissian SH, Dunn KB (2011). Anorectal melanoma: diagnosis and treatment. Dis Colon Rectum.

[140] Chin KM, Wessler B, Chew P, Lau J (2006). Genetic Tests for Cancer [Internet]. Rockville (MD): Agency for Healthcare Research and Quality (US).

[141] Llansola M, Montoliu C, Agusti A, Hernandez-Rabaza V, Cabrera-Pastor A, Malaguarnera M, Gomez-Gimenez B, Orts A, Garcia-Garcia R, Balzano T, Taoro L, Felipo V (2015). Translational research in hepatic encephalopathy: new diagnostic possibilities and new therapeutic approaches. New Horiz Transl Med.

[142] Mihajlovic M, Vlajkovic S, Jovanovic P, Stefanovic V (2012). Primary mucosal melanomas: a comprehensive review. Int J Clin Exp Pathol.

[143] Kirchoff DD, Deutsch GB, Foshag LJ, Lee JH, Sim MS, Faries MB (2016). Evolving Therapeutic Strategies in Mucosal Melanoma Have Not Improved Survival Over Five Decades. Am Surg.

